# Author Correction: Lineage tracing of acute myeloid leukemia reveals the impact of hypomethylating agents on chemoresistance selection

**DOI:** 10.1038/s41467-019-13453-2

**Published:** 2019-11-26

**Authors:** Francisco Caiado, Diogo Maia-Silva, Carolina Jardim, Nina Schmolka, Tânia Carvalho, Cláudia Reforço, Rita Faria, Branka Kolundzija, André E. Simões, Tuncay Baubec, Christopher R. Vakoc, Maria Gomes da Silva, Markus G. Manz, Ton N. Schumacher, Håkan Norell, Bruno Silva-Santos

**Affiliations:** 1 Instituto de Medicina Molecular João Lobo Antunes, Faculdade de Medicina, Universidade de Lisboa, Lisboa, Portugal; 2 Cold Spring Harbor Laboratory, Cold Spring Harbor, New York, NY USA; 3 Department of Molecular Mechanisms of Disease, University of Zurich, Zurich, Switzerland; 4 Instituto Portugues de Oncologia—Francisco Gentil, Lisbon, Portugal; 5 Department of Medical Oncology and Hematology, University Hospital Zurich and University of Zurich, Zürich, Switzerland; 6 Netherlands Cancer Institute, Amsterdam, The Netherlands

**Keywords:** Biological models, Cancer models, Cancer, Cancer stem cells, Tumour heterogeneity

Correction to: *Nature Communications* 10.1038/s41467-019-12983-z, published online 01 November 2019.

The original version of this Article omitted the following from the last sentence of the Acknowledgements:

UID/BIM/50005/2019, project funded by Fundação para a Ciência e a Tecnologia (FCT)/ Ministério da Ciência, Tecnologia e Ensino Superior (MCTES) through Fundos do Orçamento de Estado. This has now been corrected in both the PDF and HTML versions of the Article.

