# Supplementary material for: Lineage tracing of acute myeloid leukemia reveals the impact of hypomethylating agents on chemoresistance selection
Source: Nat Commun. 2019 Nov 1;10:4986. doi: 10.1038/s41467-019-12983-z (PMC6825213; doi:10.1038/s41467-019-12983-z)
Supplement: Supplementary file 1 — Supplementary Information [file 41467_2019_12983_MOESM1_ESM.pdf]

Supplementary Information

Lineage tracing of acute myeloid leukemia reveals the impact of hypomethylating agents on chemoresistance selection

Caiado *et al*

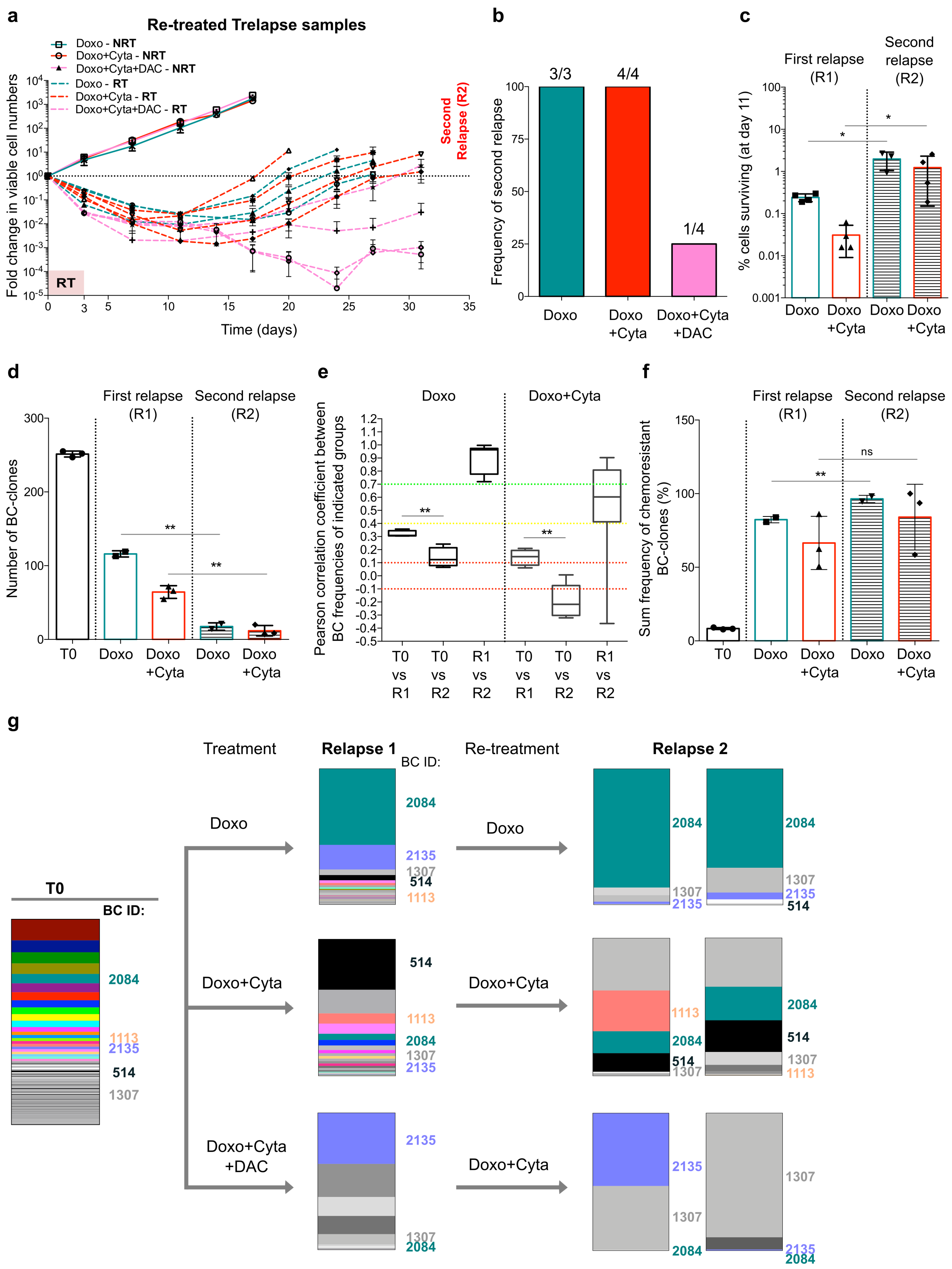

Supplementary Fig. 1 – Impact of chemotherapeutic re-exposure on Trelapse samples. (Data relative to HEL cell line) **a.** Total number of viable HEL barcoded cells between T0 and second relapse (R2), defined as a fold variation of cell number at each measured time point relatively to T0 (n=3-4), in indicated experimental groups (Doxo; Doxo+Cyta; Doxo+Cyta+DAC). Depicted are individual biological replicates (RT – re-tretment) or average values (NRT – no re-treatment). Dashed line represent fold variation of 1. **b.** Frequency of biological replicates that achieve R2. **c.** Frequency of cells surviving chemotherapy exposure (R1) and re-exposrure (R2) at equivalent day 11 after chemotherapy (re-)

exposure in indicated experimental groups (Doxo; Doxo+Cyta) (n=3-4). **d.** BC-clone numbers from different depicted experimental groups (Doxo; Doxo+Cyta) at indicated time points (T0, R1, R2)(n=2-3). **e.** Pearson correlation coefficient between BC-clonal architectures of indicated groups (Doxo; Doxo+Cyta) (n=4-9). **f.** Sum frequency of chemoresistant BC-clones in indicated experimental groups (Doxo; Doxo+Cyta) at indicated time points (T0, R1, R2) (n=2-3). **g.** Depiction of BC-clonal architecture from one representative biological replicate of each indicated experimental groups (Doxo; Doxo+Cyta; Doxo+Cyta+DAC) at indicated time points (T0, R1, R2). Chemoresistant BC-clone IDs are depicted. Graphs of mean±s.d., *P* values were determined by paired t-test .ns – not significant, * P≤0.05; ** P≤0.01. Source data are provided as a Source Data file.

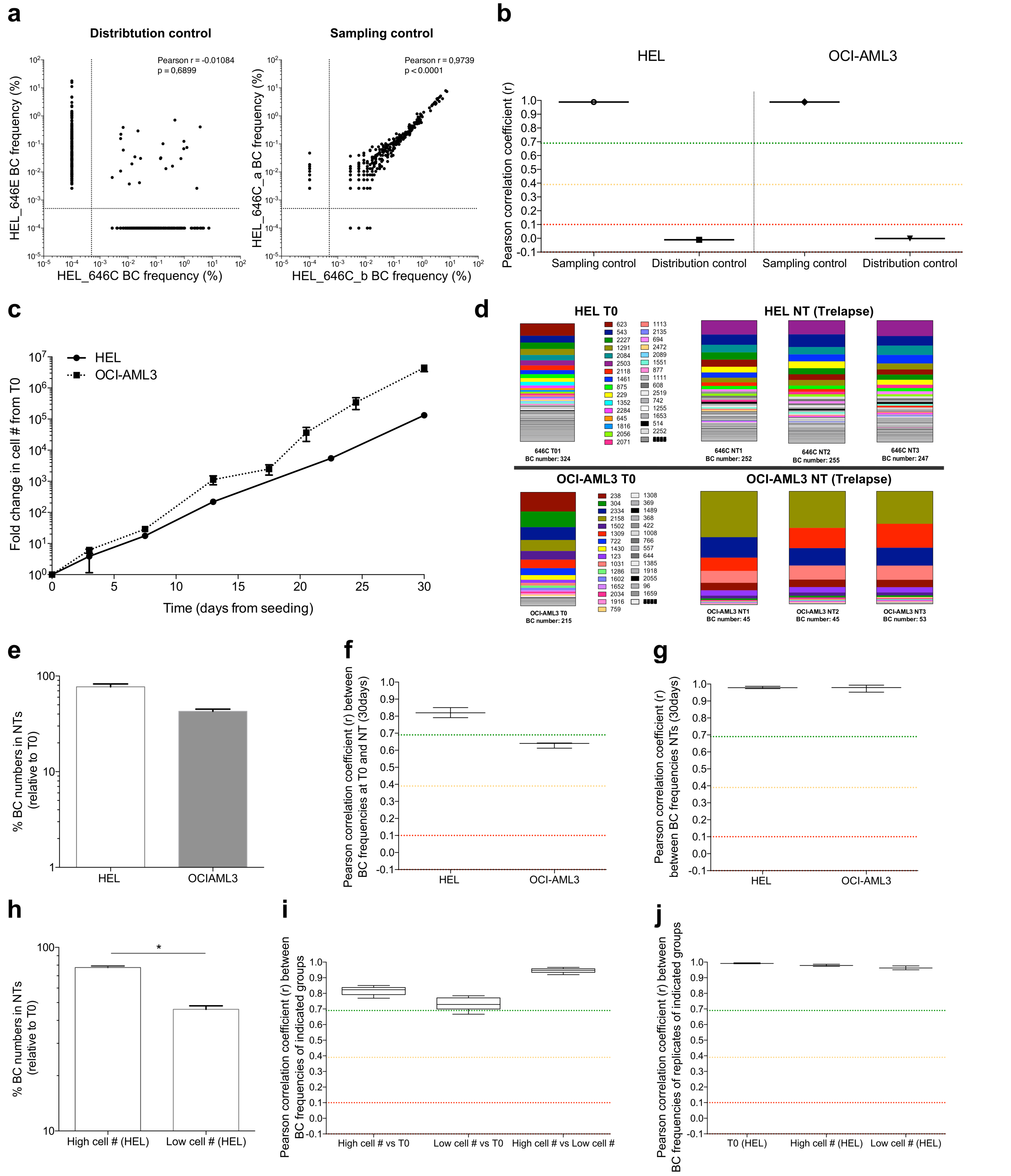

Supplementary Fig. 2 - *In vitro* BC-clonal stability of untreated barcoded hAML HEL and OCI-AML3 cell lines. a. Barcoding distribution controls performed by correlating barcode structures of two independent barcoded HEL cell lines (646C and 646E). Sampling controls performed by correlating barcode structure of two half-samples collected from 646C cell line. Pearson correlation values for sampling and distribution controls are depicted for both the HEL and OCI-AML3 cell line (**b.**) **c.** Fold change of untreated (NT) barcoded HEL and OCI-AML3 cell lines (n=3-4). **d.** Barcode structure of representative NT samples at T0 and after 30 days in culture. Each barcode-clone (BC-clone) has a fixed color-code across all samples within each cell line. **e.** Frequency of barcodes (% relative to T0) in NT

HEL and NT OCI-AML3 after 30 days continuous growth in vitro (n=3). **f.** Pearson correlation coefficient between the respective NT samples at T0 and 30 days and between replicates at day 30 (**g.**) (n=3). **h.** Frequency of barcodes (% relative to T0) in NT barcoded HEL plated at low (5000) and high (5000000) cell numbers, after 30 days of *in vitro* growth (n=3). **i.** Pearson correlation coefficient between NT samples at T0 and 30 days and between replicates at day 30 (**j.**), in low and high cell number plated barcoded HEL (n=3). Graphs of mean±s.d., *P* values were determined by t-test. ns – not significant, * P≤0.05. Source data are provided as a Source Data file.

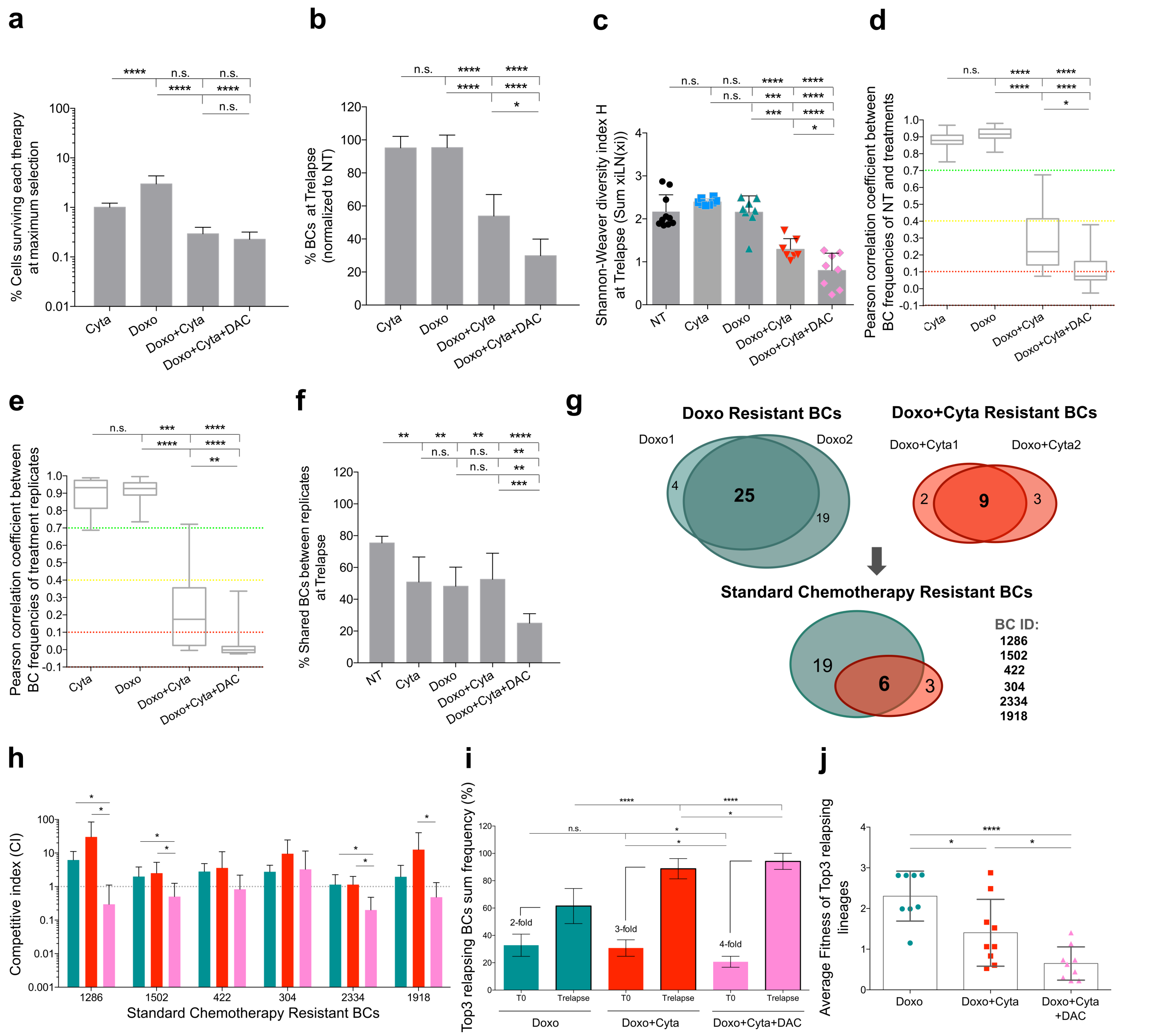

Supplementary Fig. 3 - Pre-determined set of chemotherapeutic resistant BC-clones are targeted by DAC addition leading to the selection of rarer and less fit relapsing clones. (Data relative to OCI-AML3 cell line) **a.** Fraction of cells surviving at maximum selection point in Cyta, Doxo±Cyta and Doxo+Cyta+DAC treated cells (n=8). **b.** Normalized (to NT) frequency of BC-clones detected at Trelapse (n=8-10). **c.** Shannon-Weaver diversity index H of each treatment group at Trelapse (n=8-10). **d.** Pearson correlation coefficient between BC-clonal architectures of each treatment group and NT (n=20) and between replicates within each group (**e.**) at Trelapse (n=12-15). **f.** Frequency of shared BC-clones between each group's replicates at Trelapse (n=8-10). **g.** Venn diagrams depicting the overlap of chemoresistant BC-clones, shared between all the replicates of each experiment, of 2 independent experiments for Doxo and Doxo+Cyta; as well as the overlap between these respective cores. **h.** Competitive index (CI) of each chemotherapy resistant BC-clones in the Doxo, Doxo+Cyta and Doxo+Cyta+DAC groups at Trelapse (n=8-10). A CI of 1 (dotted line) represents a similar barcode fold variation form T0 to Trelapse in the indicated condition relative to NT. **i.** Summed frequency of top 3 most dominant BC-clones at Trelapse and their matched frequency at T0. The fold variation of the summed frequency of these BC-clones between T0 and Trelapse is indicated (n=8). **j.** Average fitness of the top 3 most dominant BC-clones from Doxo, Doxo+Cyta and Doxo+Cyta+DAC groups at Trelapse (n=8). Graphs of mean±s.d., *P* values were determined by one-way ANOVA test (more than 2 groups) or t-test (2 groups). ns – not significant, * P≤0.05; ** P≤0.01; *** P≤0.001; **** P≤0.0001. Source data are provided as a Source Data file.

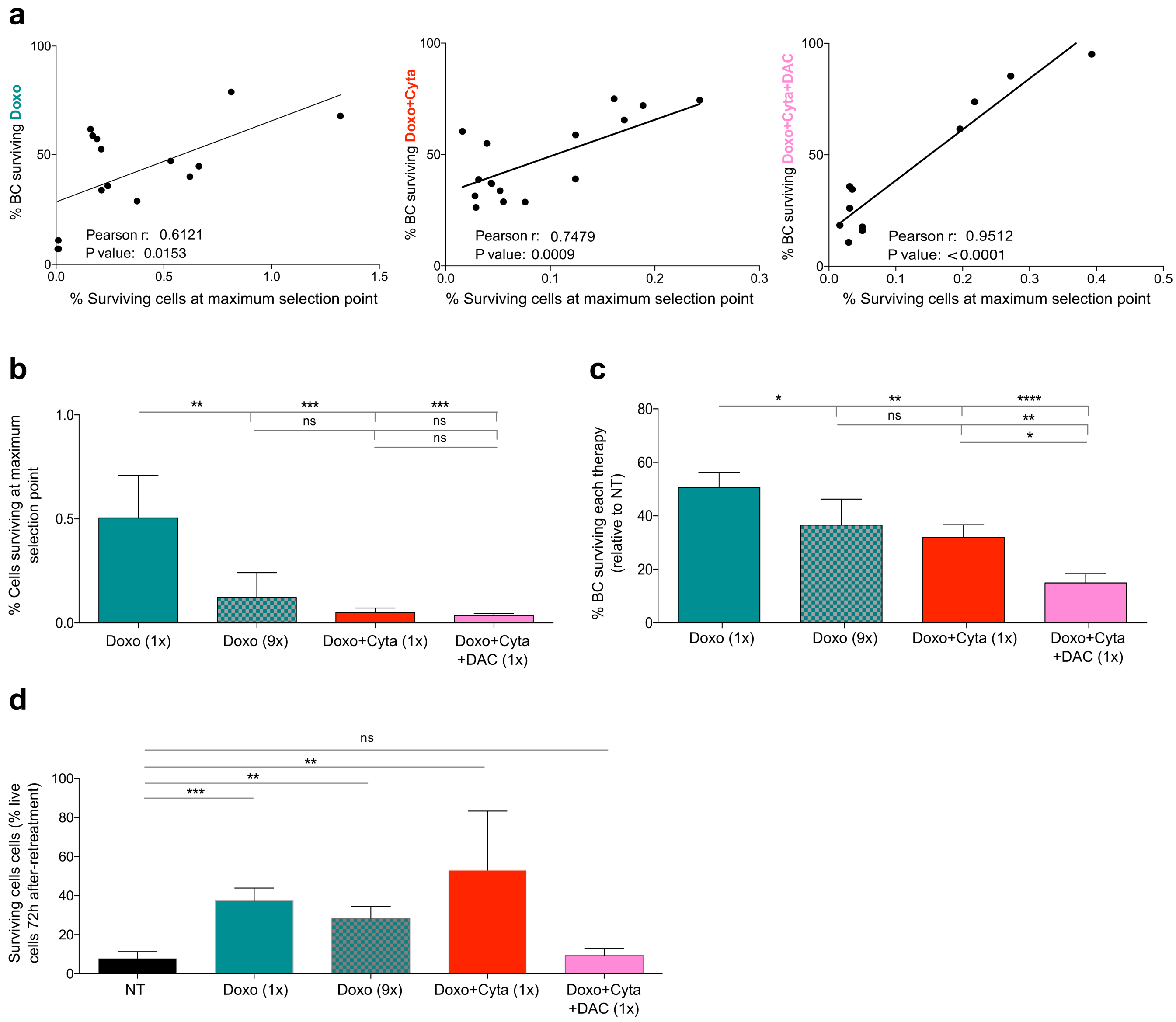

Supplementary Fig. 4 – Decitabine combination shows enhanced capacity to deplete BC-clones compared to Doxo±Cyta upon normalization of cell elimination levels. (Data relative to HEL cell line) **a.** Correlation plots between the percent of BC-clones surviving indicated therapies (Doxo, Doxo+Cyta and Doxo+Cyta+DAC) and the correspondent percent of total cells surviving at maximum selection point. Pearson correlation coefficient and associated *P*-values are indicated (n=11-14). **b.** Quantification of the percent of cells surviving at maximum selection point in indicated experimental groups (Doxo1x (1,8uM), Doxo9x (16,2uM), Doxo+Cyta1x (1,8uM doxorubicin, 6uM cytarabine), Doxo+Cyta+DAC1x (1,8uM doxorubicin, 6uM cytarabine, 0.1uM decitabine)) (n=4). **c.** Quantification of the percent of BC-clones (relative to untreated – NT - conditions) detected in indicated groups (n=4). **d.** Frequency of viable Trelapse cells from indicated groups after re-exposure to Doxo+Cyta for 72 hours (n=4). Graphs of mean±s.d., *P* values were determined by one-way ANOVA test (more than 2 groups) or t-test (2 groups). ns – not significant, * P≤0.05; ** P≤0.01; *** , P≤0.001; **** , P≤0.0001. Source data are provided as a Source Data file.

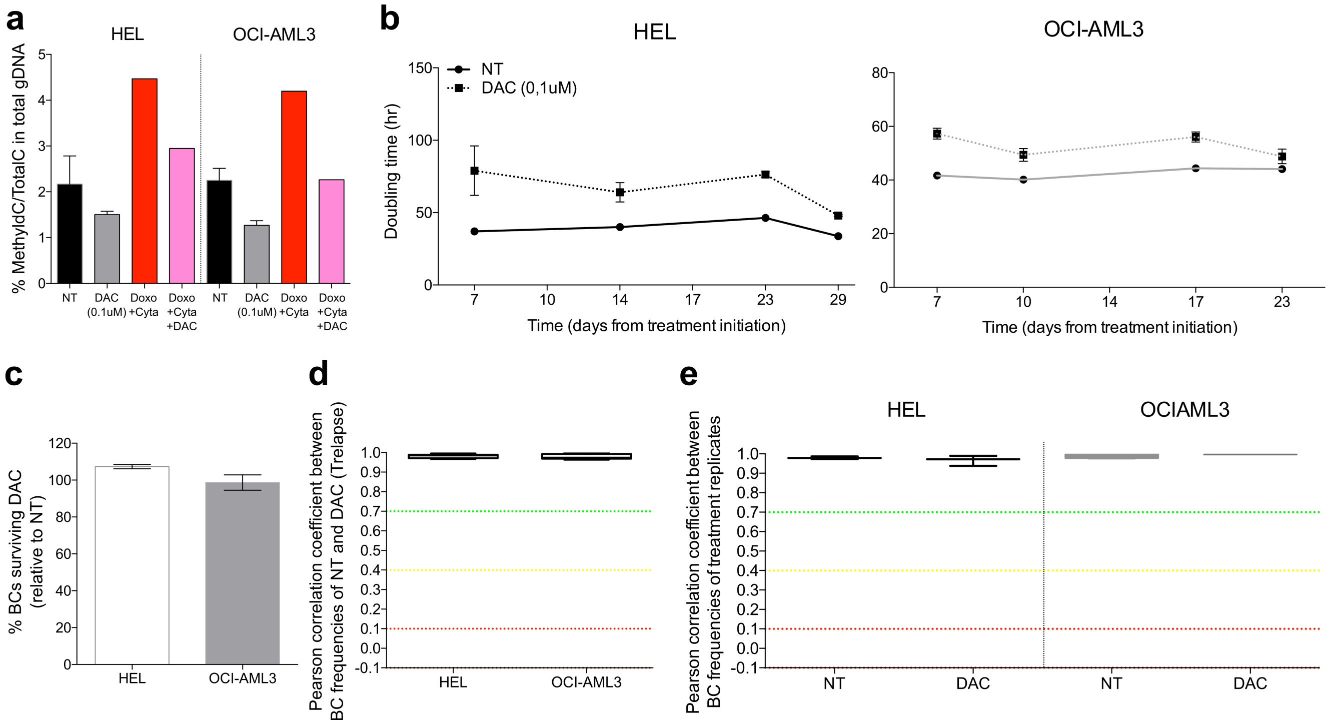

Supplementary Fig. 5 - Effect of low-dose DAC on DNA CpG methylations status, doubling time and BC-clonal composition. **a.** Normalized quantification of genomic DNA CpG methylation by MALDI mass spectrometry in HEL and OCI-AML3 cells untreated (NT), treated with low-dose DAC (0,1uM), treated with Doxo+Cyta and Doxo+Cyta+DAC regimens (n=1-2). Measurement was preformed 72h after regimen exposure. **b.** Doubling time measured for 3-4weeks after 72h exposure of HEL and OCI-AML3 cells to low-dose DAC (n=3). **c.** Frequency of BC-clones (% relative to T0) in HEL and OCI-AML3 cells at Trelapse after 72h exposure to low-dose DAC (n=3). **d.** Pearson correlation coefficient between NT and low-dose DAC exposed cells and between low-dose DAC treated replicates (**e.**) for HEL and OCI-AML3 at Trelapse (n=3). Source data are provided as a Source Data file.

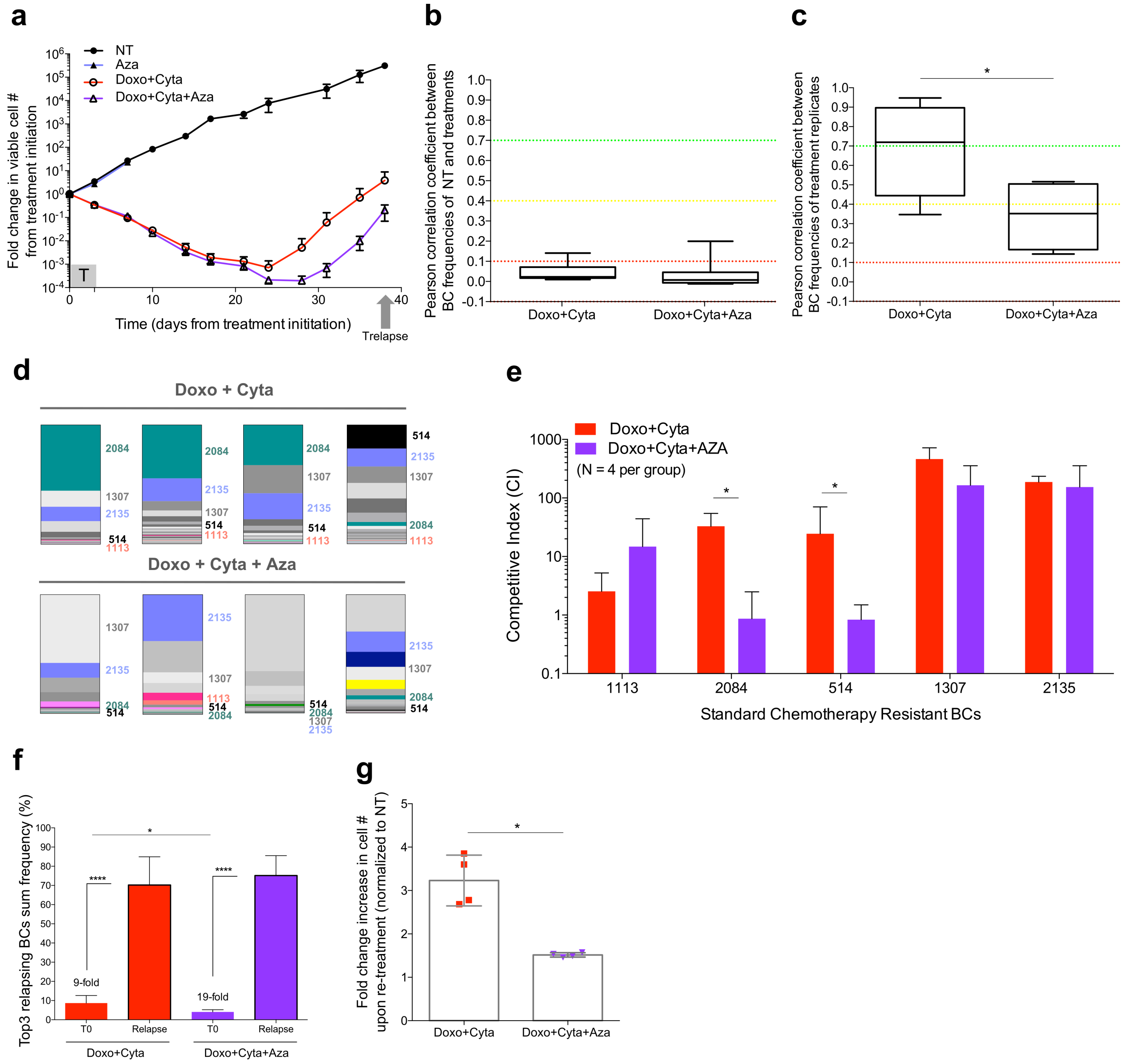

Supplementary Fig. 6 - Azacitadine combination selects for unpredictable, rarer and chemosensitive relapsing BC-clones. (Data relative to HEL cell line) **a.** Longitudinal quantification of total number of viable barcoded AML cells after NT, Aza (1uM), Doxo+Cyta and Doxo+Cyta+Aza 72h treatments (defined as a fold variation from cell number at T0) (n=3-5). **b.** Pearson correlation coefficient between BC-clonal architectures of NT and Doxo+Cyta±Aza (n=12) and between replicates in treatment groups at Trelapse (**c.**) (n=7). **d.** BC-clonal structure of representative Doxo+Cyta and Doxo+Cyta+Aza treated cells at Trelapse. Each BC-clone has a fixed color-code across all samples. Indicated barcode IDs are those of chemoresistant BC-clones. **e.** Competitive index (CI) of each chemotherapy resistant BC-clones in Doxo+Cyta and Doxo+Cyta+Aza groups at Trelapse (n=4). **f.** Summed frequency of top 3 most dominant BC-clones at Trelapse and their matched frequency at T0. The fold variation of the summed frequency of these BC-clones between T0 and Trelapse is indicated (n=4) **g.** Normalized frequency of viable Trelapse cells (Doxo+Cyta and Doxo+Cyta+Aza) after re-exposure to Doxo+Cyta for 72 hours. Graphs of mean±s.d., *P* values were determined by t-test. ns – not significant, * P≤0.05. Source data are provided as a Source Data file.

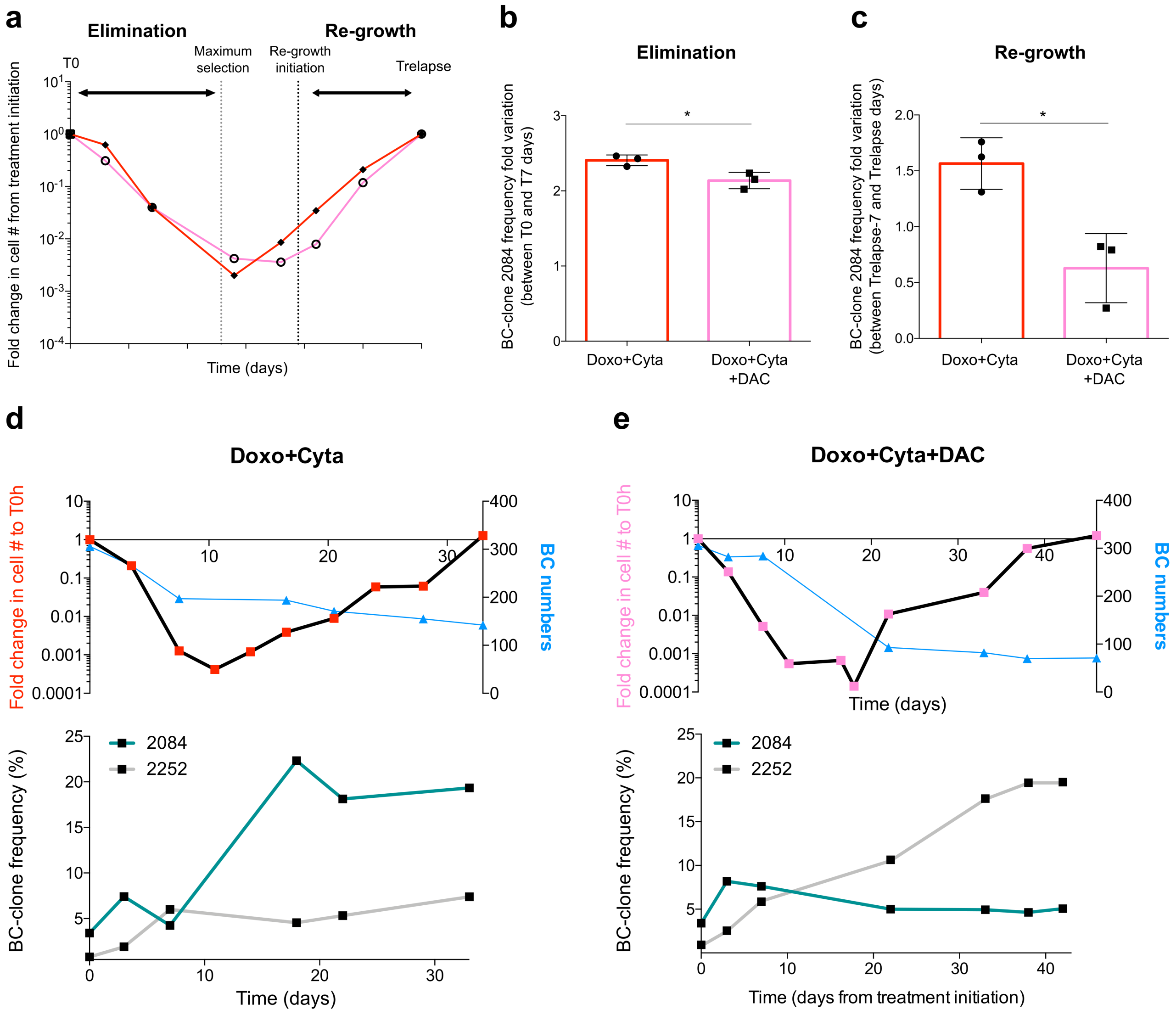

Supplementary Fig. 7 – Longitudinal assessment of decitabine combination impact on the elimination and re-growth stages of chemotherapeutic selection. (Data relative to HEL cell line) **a.** Schematic representation of the dynamic changes in cell numbers upon exposure the chemotherapeutic pressure. The elimination stage corresponds to the time between T0 and the maximum selection point (ranging from 7-12 days on average), depicted by the first dashed line. The re-growth stage corresponds to the time ranging from the first increase in cell numbers post maximum selection point, depicted by the second dashed line, until Trelapse (ranging from 15-20 days on average). **b.** Fold variation of the frequency of chemoresistant BC-clone 2084 between T0 and day 7 of the elimination stage (n=3). **c.** Fold variation of the frequency of chemoresistant BC-clone 2084 between Trelapse minus 7days and Trelapse of the re-growth stage (n=3). **d.** Upper panel – longitudinal assessment of cell number (black line, left Y-axis) and correspondent BC numbers (blue line, right Y-axis) upon Doxo+Cyta exposure. Lower panel – longitudinal assessment of BC-clones 2084 and 2252 frequency variation in the equivalent (as upper panel) experimental setup. **e.** Upper panel – longitudinal assessment of cell number (black line, left Y-axis) and correspondent BC numbers (blue line, right Y-axis) upon Doxo+Cyta+DAC exposure. Lower panel – longitudinal assessment of BC-clones 2084 and 2252 frequency variation in the equivalent (as upper panel) experimental setup. Graphs of mean±s.d., *P* values were determined by t-test. ns – not significant, * P≤0.05. Source data are provided as a Source Data file.

a

Samples	Total reads (paired-end)	Total mapped reads (% total reads)	Total mapped bases (% total bases)	Average read length (bp)	Target regions coverage - 10x (%)	Average read depth (target regions)
T0	86583489 (x2)	99.5%	99.9%	101	99.33%	166.4x
NT	30214401 (x2)	99.9%	99.9%	101	77.47%	60.7x
Doxo	36739419 (x2)	99.9%	99.9%	101	77.40%	73x
Doxo+Cyta	30887458 (x2)	99.9%	99.9%	101	60.15%	64.7x
Doxo+Cyta+DAC	29785934 (x2)	99.9%	99.9%	101	63.85%	60.3x

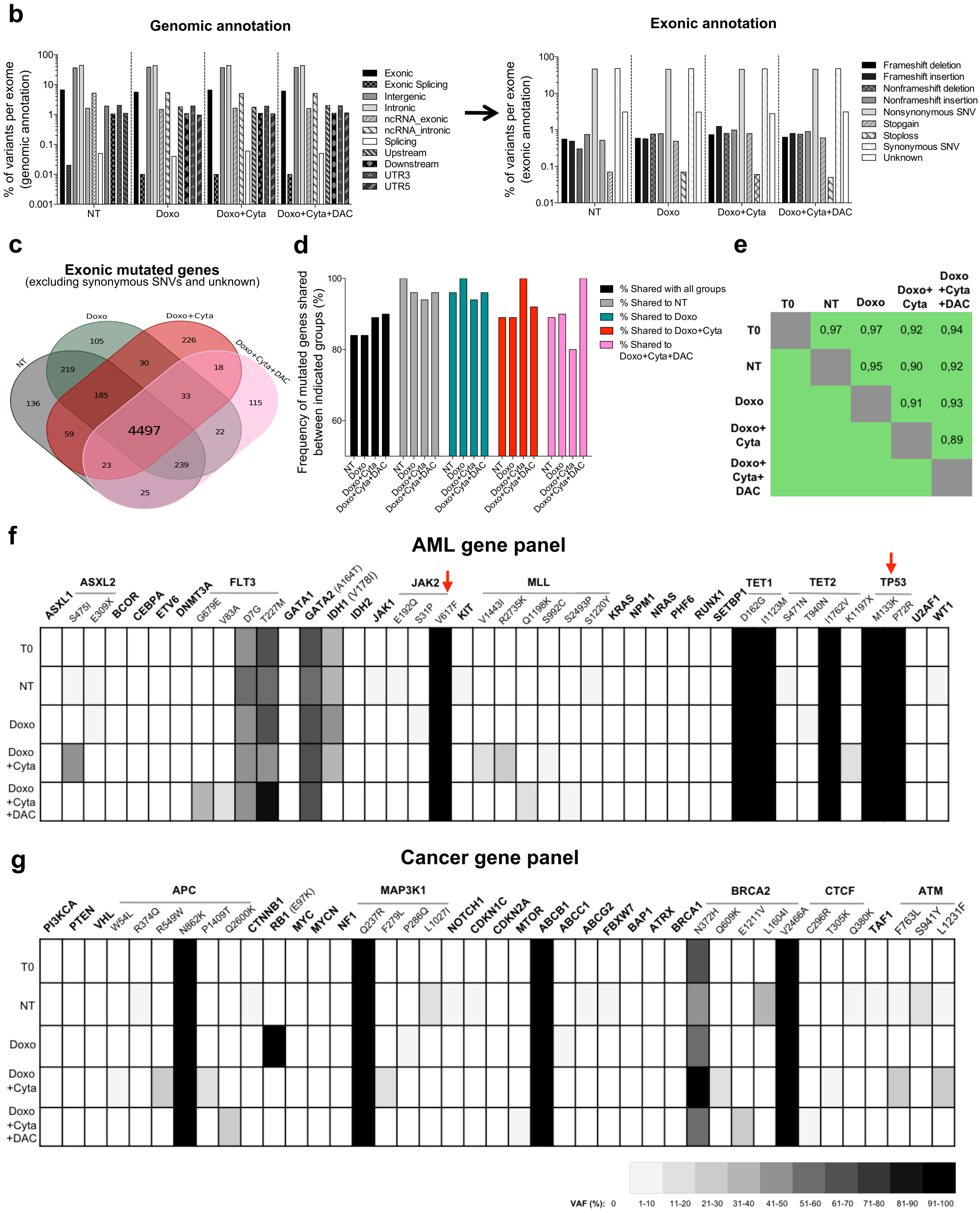

Supplementary Fig. 8 - Exome sequencing reveals overall stability of the genetic landscape of both untreated and relapsing hAML samples. (Data relative to HEL cell line) **a.** Tabular representation of initial whole exome sequencing data processing parameters for samples T0, NT, Doxo, Doxo+Cyta and Doxo+Cyta+DAC (n=1). **b.** Fraction of variants in each of the indicated genomic (left panel) and exonic (right panel) annotations, for each sample. **c.** Venn diagram depicting the intersection of sample-specific exonic mutations most likely to have direct impact on protein function (excluding synonymous and unknown variants). **d.** Fraction of sample-specific exonic mutations shared between the indicated samples. **e.** Pearson coefficient correlation matrix of exonic variant allele frequency between indicated samples. Pearson coefficient correlation is depicted for each comparison. **f.** Exonic variant allele frequencies on a defined set of genes recurrently mutated in AML and in different types of cancer (**g.**), for each sample. Variant allele frequency color code is depicted. Red arrows indicate previously described mutations in HEL cell line.

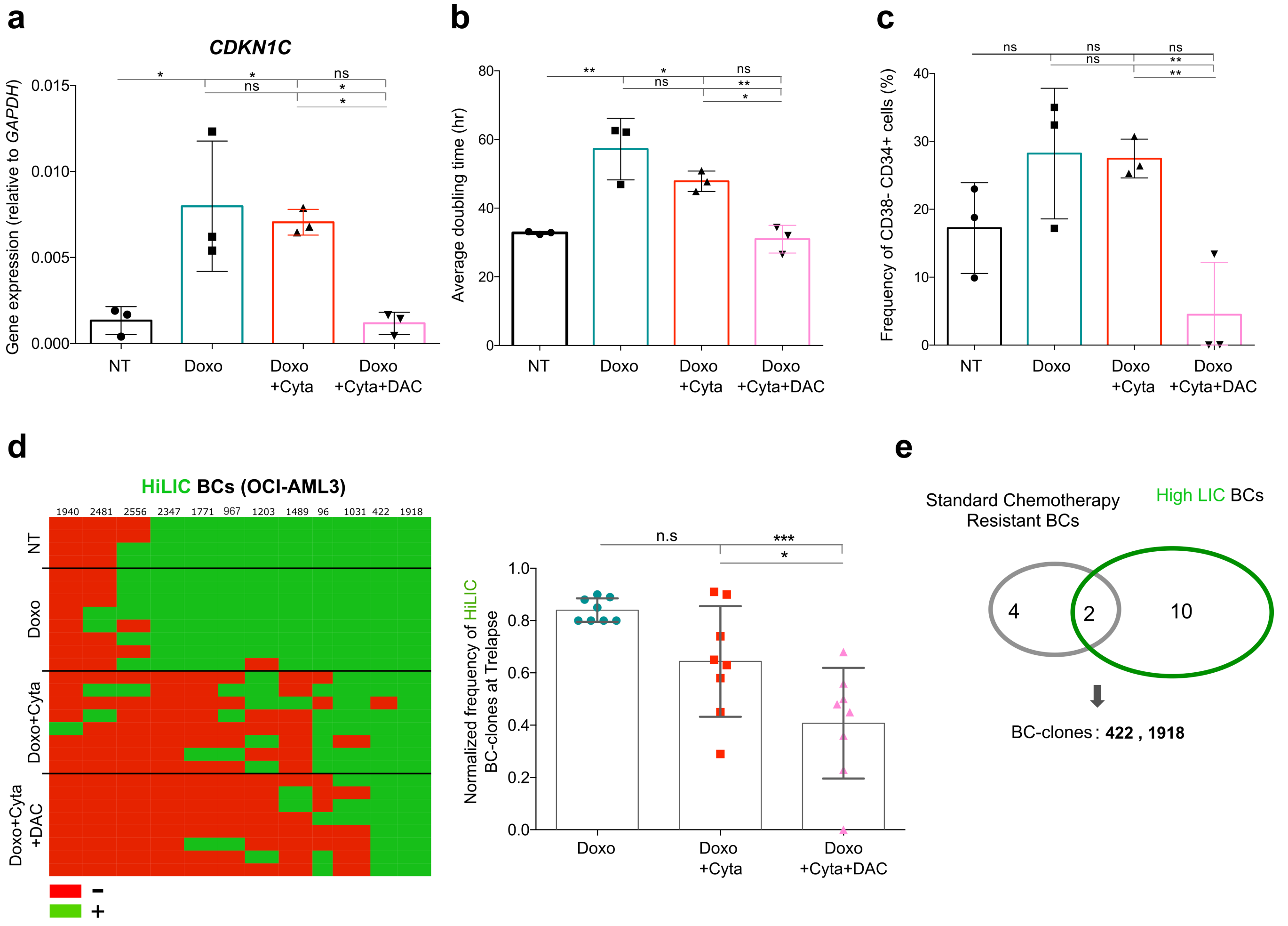

Supplementary Fig. 9 - Decitabine combination targets OCI-AML3 BC-clones with enhanced leukemia initiating potential. (Data relative to OCI-AML3 cell line) **a.** CDKN1C gene expression levels (normalized to *GAPDH* gene expression) determined by quantitative PCR in Trelapse samples (NT, Doxo±Cyta and Doxo+Cyta+DAC populations at Trelapse (n=3) **b.** Doubling time (hours) of indicated experimental groups at Trelapse (n=7). **c.** Frequency of OCI-AML3 CD38-CD34+ cells in indicated experimental groups at Trelapse (n=3). **d.** Heat-map scoring the presence (green) or absence (red) of HiL-IC BC-clones OCI-AML3 BC-clones (each column, total of 12) present in Trelapse populations from replicates of NT, Doxo±Cyta and Doxo+Cyta+DAC populations (lines). Right graph depicts the frequency of HiL-IC BC-clones present at Trelapse in Doxo±Cyta and Doxo+Cyta+DAC, normalized to the total frequency of BC-clones remaining at Trelapse relative to T0 (n=8). **e.** Venn diagram depicting the overlap across BC-clones with HiL-IC and chemoresistant properties. Graphs of mean±s.d., *P* values were determined by one-way ANOVA test. ns – not sinificant, * P≤0.05; ** P≤0.01; *** P≤0.001; **** P≤0.0001. Source data are provided as a Source Data file.

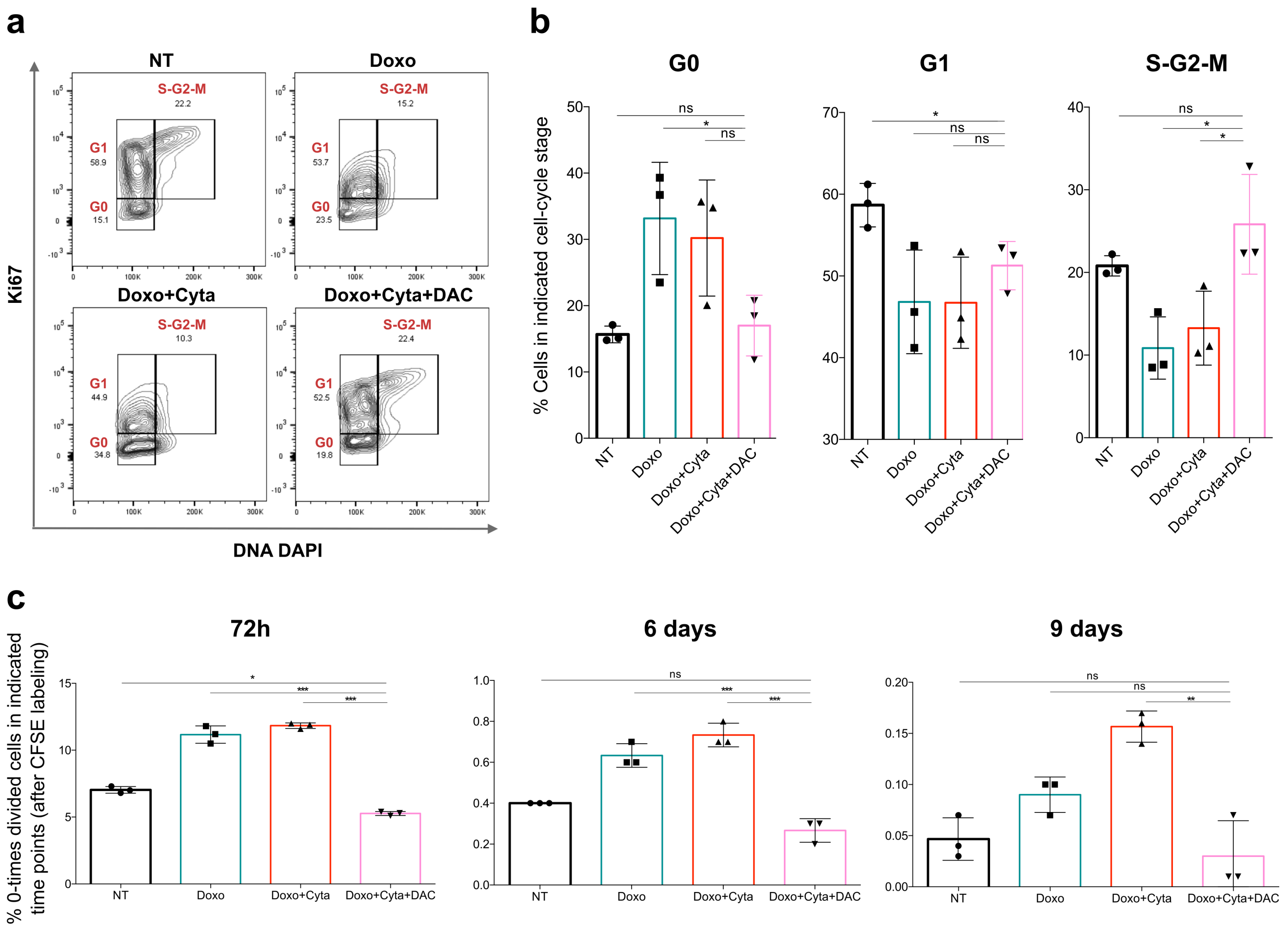

Supplementary Fig. 10 – Decitabine combination leads to relapses with increased proliferative capacity. (Data relative to HEL cell line). **a.** Cell cycle profiles determined by Ki67 and DAPI (DNA amount) staining in NT, Doxo, Doxo+Cyta and Doxo+Cyta+DAC Trelapse samples. **b.** Quantification of the frequency of cells in G0, G1 or S-G2-M phases of the cell cycle in indicated experimental groups (n=3). **c.** Quantification of the fraction of undivided cells by CFSE labeling in indicated experimental groups at 72 hours, 6 days and 9 days after CFSE labeling (undivided cell fraction is determined by CFSE staining intensity at 0 hours)(n=3). Graphs of mean±s.d., *P* values were determined by t-test. ns – not significant, * P≤0.05; ** P≤0.01; *** P≤0.001; **** P≤0.0001. Source data are provided as a Source Data file.

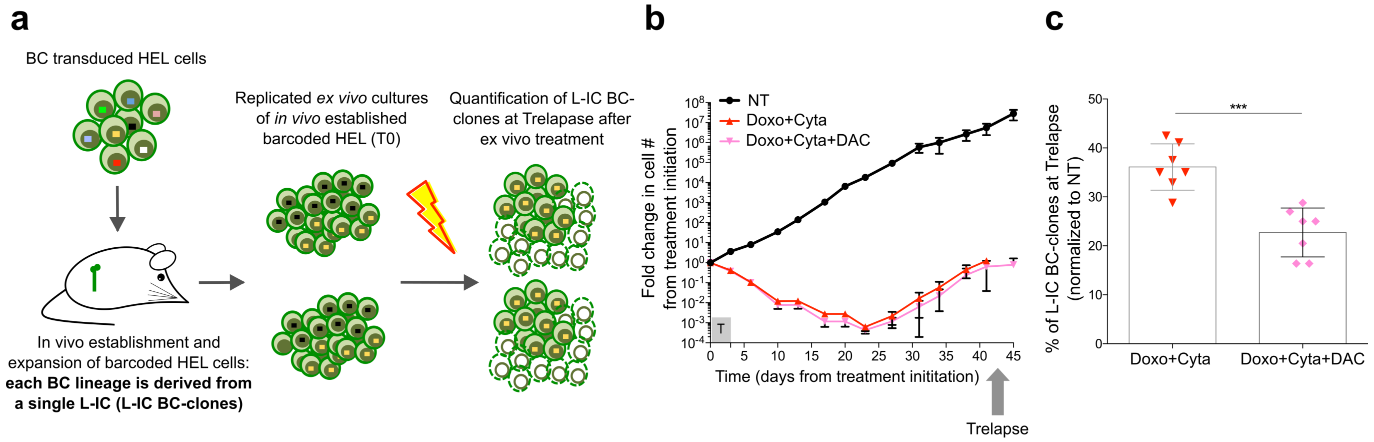

Supplementary Fig. 11 - Decitabine combination shows enhanced targeting of *in vivo* derived L-IC BC-clones. a. Schematic diagram depicting *in vivo* establishment of freshly barcoded HEL cells (prior to *in vitro* expansion) and posterior ex vivo treatment with Doxo±Cyta and Doxo+Cyta+DAC. In this system each BC-clones derive from a single L-IC that established in NRGS mice and generated a L-IC BC-clone. **b.** Total number of viable *in vivo* established barcoded HEL cells between T0 and Trelapse (defined as a fold variation from cell number at T0) upon *ex vivo* exposure to NT, Doxo±Cyta and Doxo+Cyta+DAC (n=7). **c.** Frequency of L-IC BC-clones (normalized to NT) present at Trelapse after *ex vivo* exposure to Doxo±Cyta and Doxo+Cyta+DAC (n=7). Graphs of mean±s.d., *P* values were determined by one-way ANOVA test. ns – not significant, * P≤0.05; ** P≤0.01; *** P≤0.001; **** P≤0.0001. Source data are provided as a Source Data file.

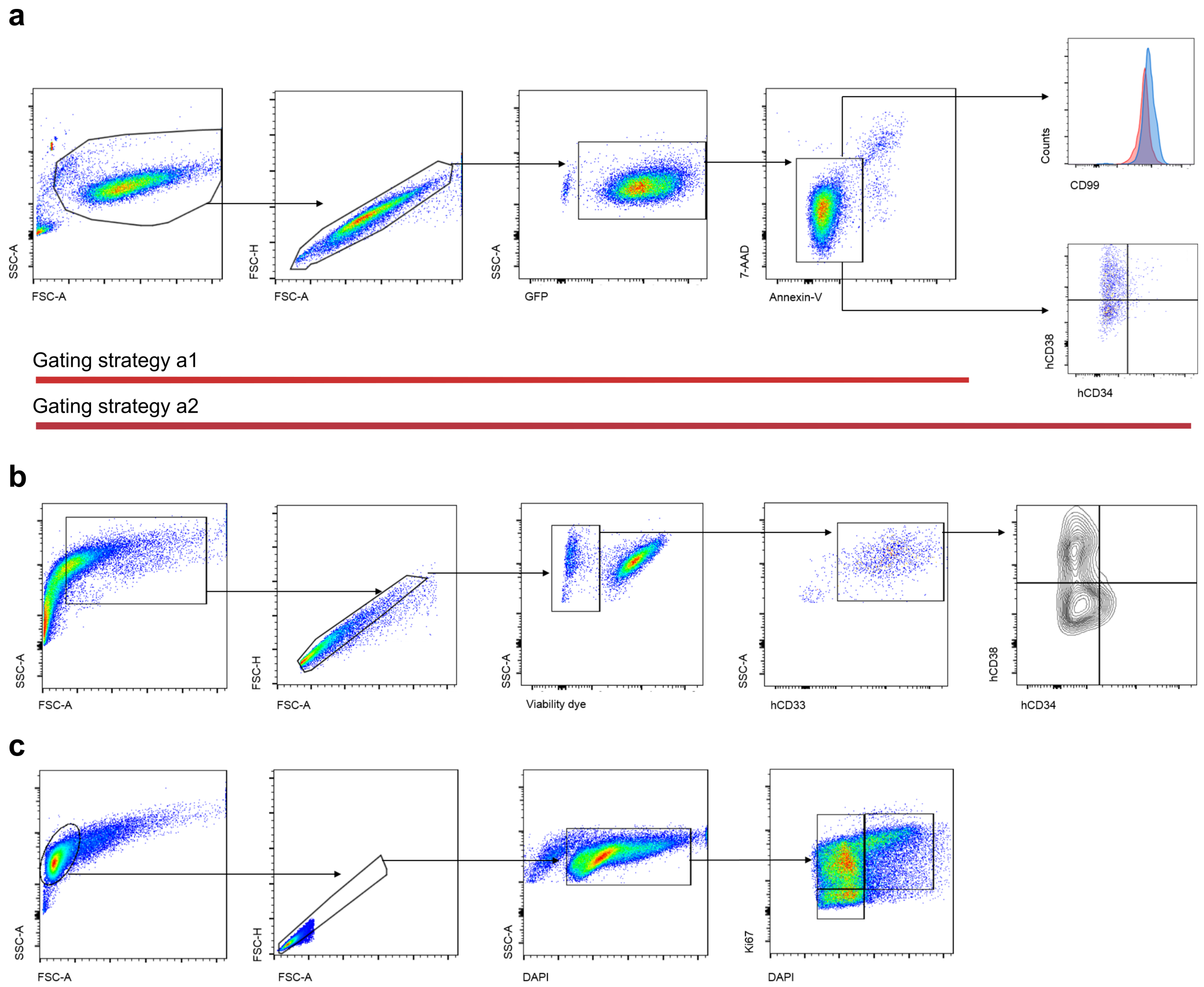

Supplementary Fig. 12 – Gating strategies. a. Gating strategy to quantify viable cell numbers of cell HEL and OCIAML-3. Strategy a1 was used in cell quantifications depicted in figure 1b and c, figure 4e and f, figure 6b and d, supplementary figures 1a, 3a, 4b, 6a and g, 7d and e, 11b. Strategy a1 precedes panel shown in figure4i. Strategy a2 used to quantify CD99 (upper) or CD38- CD34+ (lower) leukemia stem cell populations. This strategy was used in quantifications depicted in figure 4c, 6e and supplementary figure 9c. **b.** Gating strategy to quantify CD33+ CD38- CD34+ leukemia stem cell populations in primary human AML samples, relative to figure 6h. **c.** Gating strategy to quantify cell cycle stages. This strategy was used in supplementary figure 10a and b.

Supplementary Table 1 – AML patient characteristics (related to figure 6)

Patient Number	Age	Sex	FAB
1	87	M	M4
3	73	F	M1
11	80	M	M5a
12	45	F	M4
15	70	M	M1
20	62	M	M2
25	72	F	M2
38	58	F	M4
