# Supplementary material for: Lineage tracing of acute myeloid leukemia reveals the impact of hypomethylating agents on chemoresistance selection
Source: Nat Commun. 2019 Nov 1;10:4986. doi: 10.1038/s41467-019-12983-z (PMC6825213; doi:10.1038/s41467-019-12983-z)
Supplement: Supplementary file 3 — Description of Additional Supplementary Files [file 41467_2019_12983_MOESM3_ESM.pdf]

Description of Additional Supplementary Files

File Name: Supplementary Data 1

Description: File containing differential gene expression levels (fold change > 2 and adjusted p-value < 0.01) between indicated groups. Data is derived from RNA-Seq data analysis as indicated in the materials and methods section.
