# Supplementary material for: Lineage tracing of acute myeloid leukemia reveals the impact of hypomethylating agents on chemoresistance selection
Source: Nat Commun. 2019 Nov 1;10:4986. doi: 10.1038/s41467-019-12983-z (PMC6825213; doi:10.1038/s41467-019-12983-z)
Supplement: Supplementary file 5 — Reporting Summary [file 41467_2019_12983_MOESM5_ESM.pdf]

nature research

Corresponding author(s): Bruno Silva-Santos, Hakan Norell, Francisco Caiado

Last updated by author(s): Sep 20, 2019

# Reporting Summary

Nature Research wishes to improve the reproducibility of the work that we publish. This form provides structure for consistency and transparency in reporting. For further information on Nature Research policies, see Authors & Referees and the Editorial Policy Checklist.

## Statistics

For all statistical analyses, confirm that the following items are present in the figure legend, table legend, main text, or Methods section.

| n/a | Confirmed | |
|---|---|---|
| ☐ | ☒ | The exact sample size (*n*) for each experimental group/condition, given as a discrete number and unit of measurement |
| ☐ | ☒ | A statement on whether measurements were taken from distinct samples or whether the same sample was measured repeatedly |
| ☐ | ☒ | The statistical test(s) used AND whether they are one- or two-sided<br>*Only common tests should be described solely by name; describe more complex techniques in the Methods section.* |
| ☒ | ☐ | A description of all covariates tested |
| ☐ | ☒ | A description of any assumptions or corrections, such as tests of normality and adjustment for multiple comparisons |
| ☐ | ☒ | A full description of the statistical parameters including central tendency (e.g. means) or other basic estimates (e.g. regression coefficient) AND variation (e.g. standard deviation) or associated estimates of uncertainty (e.g. confidence intervals) |
| ☐ | ☒ | For null hypothesis testing, the test statistic (e.g. *F*, *t*, *r*) with confidence intervals, effect sizes, degrees of freedom and *P* value noted<br>*Give P values as exact values whenever suitable.* |
| ☒ | ☐ | For Bayesian analysis, information on the choice of priors and Markov chain Monte Carlo settings |
| ☒ | ☐ | For hierarchical and complex designs, identification of the appropriate level for tests and full reporting of outcomes |
| ☐ | ☒ | Estimates of effect sizes (e.g. Cohen's *d*, Pearson's *r*), indicating how they were calculated |

*Our web collection on statistics for biologists contains articles on many of the points above.*

## Software and code

Policy information about availability of computer code

| | |
|---|---|
| Data collection | No software was used. |
| Data analysis | GraphPad Prism 7 was used for statistical analysis |

For manuscripts utilizing custom algorithms or software that are central to the research but not yet described in published literature, software must be made available to editors/reviewers. We strongly encourage code deposition in a community repository (e.g. GitHub). See the Nature Research guidelines for submitting code & software for further information.

## Data

Policy information about availability of data

All manuscripts must include a data availability statement. This statement should provide the following information, where applicable:

- Accession codes, unique identifiers, or web links for publicly available datasets
- A list of figures that have associated raw data
- A description of any restrictions on data availability

The authors declare that the data supporting the findings of this study are available within the article and its Supplementary Information Files. All files are available from the corresponding authors on reasonable request.

# Field-specific reporting

Please select the one below that is the best fit for your research. If you are not sure, read the appropriate sections before making your selection.

☒ Life sciences ☐ Behavioural & social sciences ☐ Ecological, evolutionary & environmental sciences

For a reference copy of the document with all sections, see nature.com/documents/nr-reporting-summary-flat.pdf

# Life sciences study design

All studies must disclose on these points even when the disclosure is negative.

| | |
|---|---|
| Sample size | Sample size was chosen depending on the experimental approaches used and estimated based on our experience. No statistical method was used to predetermine sample size. |
| Data exclusions | No data was excluded. |
| Replication | The shown results could successfully and reliably be replicated. |
| Randomization | The replicates were derived from the same initial cell line, therefore no randomization was necessary for the in vitro treatments. Mice carrying different levels of leukemic burden were randomly assigned to experimental groups using an excel randomization function. |
| Blinding | Analysis blinding was preformed by assignment of unique identification codes to each sample which were unknown to the observer. |

# Reporting for specific materials, systems and methods

We require information from authors about some types of materials, experimental systems and methods used in many studies. Here, indicate whether each material, system or method listed is relevant to your study. If you are not sure if a list item applies to your research, read the appropriate section before selecting a response.

## Materials & experimental systems

| n/a | Involved in the study | |
|---|---|---|
| ☐ | ☒ | Antibodies |
| ☐ | ☒ | Eukaryotic cell lines |
| ☒ | ☐ | Palaeontology |
| ☐ | ☒ | Animals and other organisms |
| ☐ | ☒ | Human research participants |
| ☒ | ☐ | Clinical data |

## Methods

| n/a | Involved in the study | |
|---|---|---|
| ☒ | ☐ | ChIP-seq |
| ☐ | ☒ | Flow cytometry |
| ☒ | ☐ | MRI-based neuroimaging |

## Antibodies

| | |
|---|---|
| Antibodies used | AnnexinV-APC (InvitrogenTM, NY, USA) ; anti-human CD34-APC (Biolegend, USA), anti-human CD38-PE (Biolegend, USA) and anti-human CD99-PE (Biolegend, USA); anti-human CD33-APC (Biolegend, USA) |
| Validation | No antibody validation was preformed since used antibodies are well established in multiple studies cited by manufacturer. Negative controls for each antibody were always included in staining panels to control for antibody unspecificity. |

## Eukaryotic cell lines

Policy information about cell lines

| | |
|---|---|
| Cell line source(s) | ATCC |
| Authentication | Cells were newly acquired to ATCC. No further validation was preformed. |
| Mycoplasma contamination | Cell lines were tested for micoplasma contamination prior to the study and results were negative. |
| Commonly misidentified lines (See ICLAC register) | Not used |

## Animals and other organisms

Policy information about studies involving animals; ARRIVE guidelines recommended for reporting animal research

| | |
|---|---|
| Laboratory animals | Mus musculus ; NOD-Rag1-/-γc-/- (NRGS, 024099) were obtained from the Jackson Laboratories (USA) ; 8-10 weeks old male and females |
| Wild animals | The study did not involve wild animals. |
| Field-collected samples | The study did not involve samples collected from the field. |

Ethics oversight | Ethics commitee of Instituto de Medicina Molecular, Lisbon, Portugal

Note that full information on the approval of the study protocol must also be provided in the manuscript.

# Human research participants

Policy information about studies involving human research participants

Population characteristics | 8 patient samples (human biospecimens) were used. Patients age: 45-87 ; 4 Males , 4 Females.

Recruitment | Samples were biobanked after colection at Portuguese Institute of Oncology by assigned hematologists.

Ethics oversight | Ethics commitee of Portuguese Institute of Oncology, Lisbon, Portugal

Note that full information on the approval of the study protocol must also be provided in the manuscript.

# Flow Cytometry

## Plots

Confirm that:

- ☒ The axis labels state the marker and fluorochrome used (e.g. CD4-FITC).
- ☒ The axis scales are clearly visible. Include numbers along axes only for bottom left plot of group (a 'group' is an analysis of identical markers).
- ☒ All plots are contour plots with outliers or pseudocolor plots.
- ☒ A numerical value for number of cells or percentage (with statistics) is provided.

## Methodology

Sample preparation | Cells were washed in PBS + 2% FBS, centrifuged at 350g for 5 minutes and incubated with indicated antibodies at specific dilutions (depending on antibody).

Instrument | LSR II Fortessa BD

Software | FlowJo V10.

Cell population abundance | Abundances are depicted in the manuscript. For human CD38- CD34+ populations, the frequency ranged from 0,1%-5% of the total population of live cells.

Gating strategy | Gating strategies depicted in Supplementary Figure 12.

☒ Tick this box to confirm that a figure exemplifying the gating strategy is provided in the Supplementary Information.
